# Supplementary material for: Impact of High-Fat Diet and Exercise on Bone and Bile Acid Metabolism in Rats
Source: Nutrients. 2024 Jun 2;16(11):1744. doi: 10.3390/nu16111744 (PMC11174439; doi:10.3390/nu16111744)
Supplement: Supplementary file 1 [file nutrients-16-01744-s001.zip › nutrients-3030282-supplementary.docx]

Supplementary Materials

Impact of High-Fat Diet and Exercise on Bone and Bile Acid Metabolism in Rats

Nerea Alonso ^1^, Gunter Almer ^1^, Maria Donatella Semeraro ^1^, Giovanny Rodriguez-Blanco ^1,2^, Günter Fauler ^1^,
Ines Anders ^3^, Gerald Ritter ^3^, Annika vom Scheidt ^4^, Niels Hammer ^4,5,6^, Hans-Jürgen Gruber ^1^
and Markus Herrmann ^1,^*

^1^ Clinical Institute for Medical and Chemical Laboratory Diagnostics (CIMCL), Medical University of Graz, 8036 Graz, Austria

^2^ LKH-Universitätsklinikum Graz, 8036 Graz, Austria

^3^ Division of Biomedical Research, Medical University of Graz, 8036 Graz, Austria

^4^ Department of Anatomy, Medical University of Graz, 8036 Graz, Austria

^5^ Department of Orthopaedic and Trauma Surgery, University of Leipzig, 04103 Leipzig, Germany

^6^ Division of Biomechatronics, Fraunhofer Institute for Machine Tools and Forming Technology,
01187 Dresden, Germany

***** Correspondence: markus.herrmann@medunigraz.at; Tel.: +43-(0)-316-385-131-45

**Table S1.** List of bile acids analysed in serum and stool in the study.

| **Bile acid** | **Type** | **Species** | **Modification** |
| --- | --- | --- | --- |
| Cholic acid (CA) | Primary bile acid | Human and murine | - |
| Taurocholic acid (TCA) | Primary bile acid | Human and murine | Taurine |
| Glycocholic acid (GCA) | Primary bile acid | Human and murine | Glycine |
| Chenodeoxycholic acid (CDCA) | Primary bile acid | Human and murine | - |
| Taurochenodeoxycholic acid (TCDCA) | Primary bile acid | Human and murine | Taurine |
| Glycochenodeoxycholic acid (GCDCA) | Primary bile acid | Human and murine | Glycine |
| Deoxycholic acid (DCA) | Secondary bile acid | Human and murine | - |
| Taurodeoxycholic acid (TDCA) | Secondary bile acid | Human and murine | Taurine |
| Glycodeoxycholic acid (GDCA) | Secondary bile acid | Human and murine | Glycine |
| Ursodeoxycholic acid (UDCA) | Secondary bile acid | Human and murine | - |
| Tauroursodeoxycholic acid (TUDCA) | Secondary bile acid | Human and murine | Taurine |
| Glycoursodeoxycholic acid (GUDCA) | Secondary bile acid | Human and murine | Glycine |
| Lithocholic acid (LCA) | Secondary bile acid | Human and murine | - |
| Taurolithocholic acid (TLCA) | Secondary bile acid | Human and murine | Taurine |
| Glycolithocholic acid (GLCA) | Secondary bile acid | Human and murine | Glycine |
| α-muricholic acid (aMUA) | Primary bile acid | Murine only | - |
| Tauro-α-muricholic acid (TaMUA) | Primary bile acid | Murine only | Taurine |
| Glyco-α-muricholic acid (GaMUA) | Primary bile acid | Murine only | Glycine |
| β-muricholic acid (bMUA) | Primary bile acid | Murine only | - |
| Tauro-β-muricholic acid (TbMUA) | Primary bile acid | Murine only | Taurine |
| Glyco-β-muricholic acid (GbMUA) | Primary bile acid | Murine only | Glycine |
| γ-muricholic acid (gMUA) | Primary bile acid | Murine only | - |
| Tauro-γ-muricholic acid (TgMUA) | Primary bile acid | Murine only | Taurine |
| Glyco-γ-muricholic acid (GgMUA) | Primary bile acid | Murine only | Glycine |
| ω-muricholic acid (oMUA) | Secondary bile acid | Murine only | - |
| Tauro-ω-muricholic acid (ToMUA) | Secondary bile acid | Murine only | Taurine |
| Hyodeoxycholic acid (HDCA) | Secondary bile acid | Human and murine | - |
| Taurohyodeoxycholic acid (THDCA) | Secondary bile acid | Human and murine | Taurine |
| Glycohyodeoxycholic acid (GHDCA) | Secondary bile acid | Human and murine | Glycine |


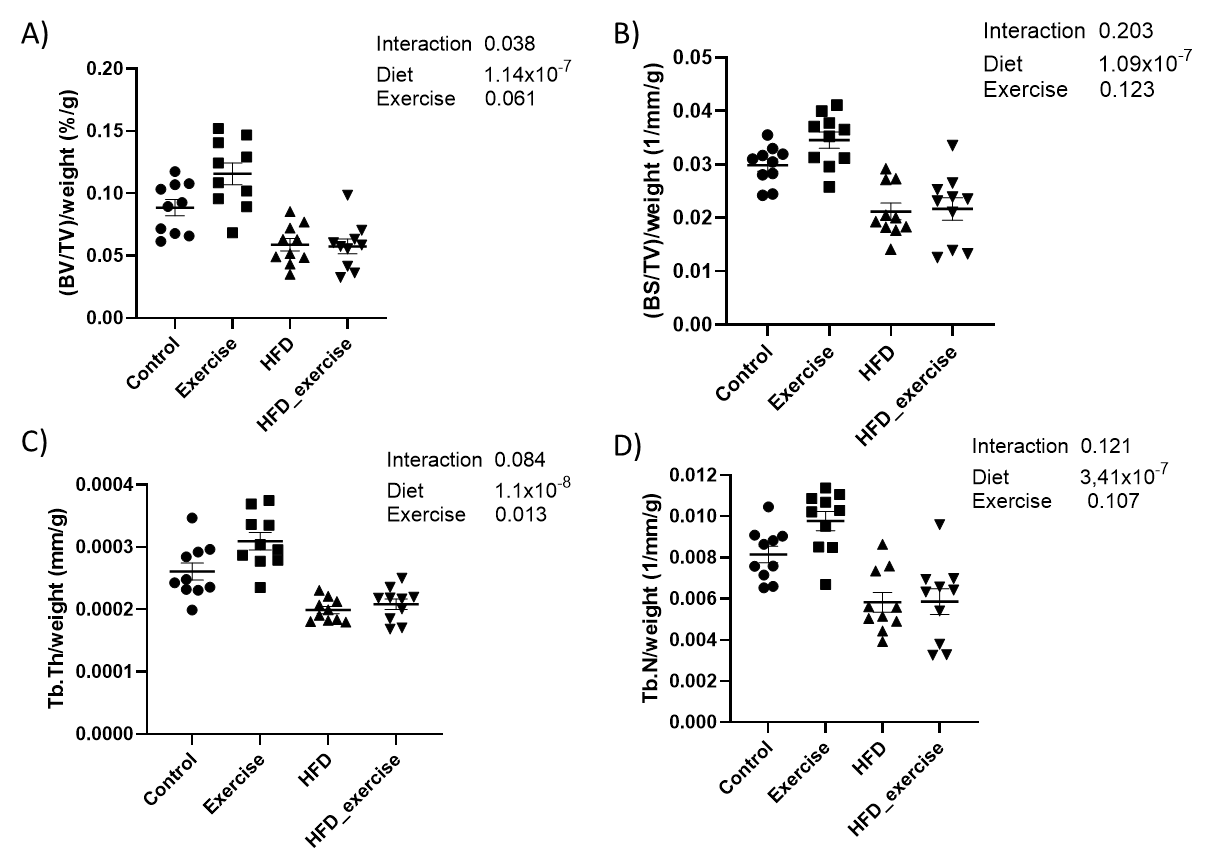


**Scheme 1.** Trabecular bone parameters in each group analysed by microCT: A) bone volume fraction (BV/TV); B) bone surface density (BS/TV); C) trabecular thickness (Tb.Th); D) trabecular number (Tb.N). Each data point corresponds to one animal and lines show mean ± SEM. All the parameters are corrected by weight. Two-way ANOVA statistics are shown per graph.


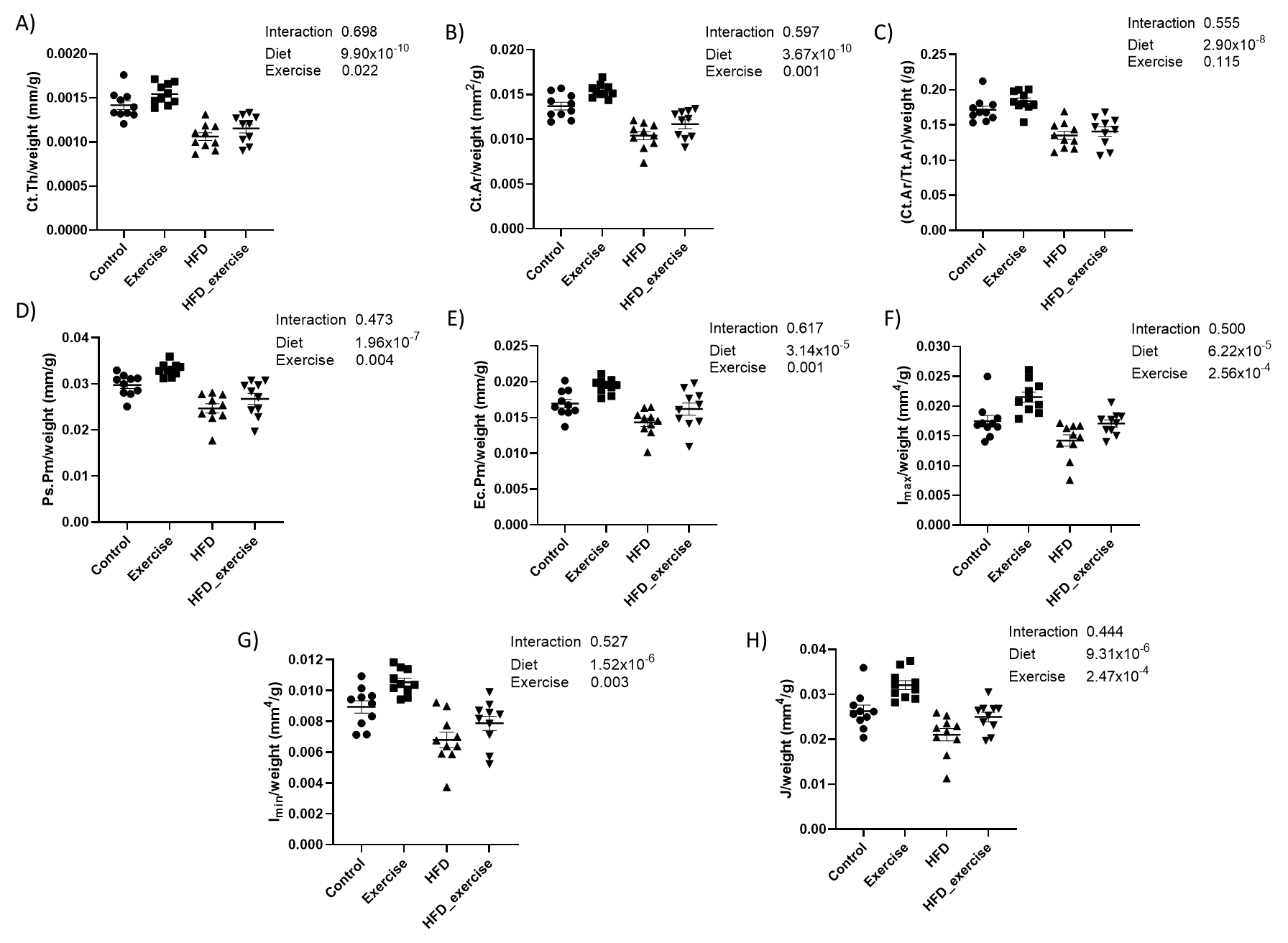


**Figure S2.** Cortical bone parameters in each group analysed by microCT: A) cortical thickness (Ct.Th); B) cortical area (Ct.Ar); C) cortical area fraction (Ct.Ar/Tt.Ar); D) periosteal perimeter (Ps.Pm); E) endocortical perimeter (Ec.Pm); F) maximal inertia (I_max_); G) minimal inertia (I_min_); H) polar moment of inertia (J). Each data point corresponds to one animal and lines show mean ± SEM. All the parameters are corrected by weight. Two-way ANOVA statistics are shown per graph.

**Table S2.** List of ratios and bile acid groups analysed in serum and stool.

| **Group** | **Composition** |
| --- | --- |
| Total BAs | Sum of 29 BAs (Suppl Table S2) |
| Free BAs | CA, CDCA, DCA, UDCA, LCA, αMUA, βMUA, γMUA, ωMUA, HDCA |
| Conjugated BAs | T/G-CA, T/G-CDCA, T/G-DCA, T/G-UDCA, T/G-LCA, T/G-αMUA, T/G-βMUA, T/G-γMUA, TωMUA, T/G-HDCA |
| Primary BAs | As described in Suppl Table S2 |
| Secondary BAs | As described in Suppl Table S2 |
| 12-α-hydroxylated BAs | CA, TCA, GCA, DCA, TDCA, GDCA |
| Non 12-α-hydroxylated | CDCA, TCDCA, GCDCA, UDCA, TUDCA, GUDCA, LCA TLCA, GLCA, αMUA, TαMUA , GαMUA, βMUA, TβMUA, GβMUA, γMUA, TγMUA, GγMUA, ωMUA, TωMUA, HDCA, THDCA, GHDCA |
|  |  |
| **Ratios** | TCA/CA |
|  | GCA/CA |
|  | TCA / GCA |
|  | CA / CDCA |
|  | TCDCA / CDCA |
|  | GCDCA / CDCA |
|  | TCDCA / GCDCA |
|  | DCA / CA |
|  | TDCA / DCA |
|  | GDCA / DCA |
|  | TDCA / GDCA |
|  | LCA / CDCA |
|  | TLCA / LCA |
|  | GLCA / LCA |
|  | TLCA / GLCA |
|  | UDCA / CDCA |
|  | TUDCA / UDCA |
|  | GUDCA / UDCA |
|  | TUDCA / GUDCA |
|  | TαMUA / αMUA |
|  | αMUA / CDCA |
|  | GαMUA / αMUA |
|  | TαMUA / GαMUA |
|  | TβMUA / βMUA |
|  | GβMUA / βMUA |
|  | TβMUA / GβMUA |
|  | βMUA / UDCA |
|  | TγMUA / γMUA |
|  | GγMUA / γMUA |
|  | TγMUA / GγMUA |
|  | THDCA / HDCA |
|  | TωMUA / ωMUA |
|  | CA (stool) / CA (serum) |
|  | TCA (stool) / TCA (serum) |
|  | GCA (stool) / GCA (serum) |
|  | CDCA (stool) / CDCA (serum) |
|  | TCDCA (stool) / TCDCA (serum) |
|  | GCDCA (stool) / GCDCA (serum) |
|  | DCA (stool) / DCA (serum) |
|  | TDCA (stool) / TDCA (serum) |
|  | GDCA (stool) / GDCA (serum) |
|  | LCA (stool) / LCA (serum) |
|  | TLCA (stool) / TLCA (serum) |
|  | GLCA(stool) / GLCA (serum) |
|  | UDCA (stool) / UDCA (serum) |
|  | TUDCA (stool) / TUDCA (serum) |
|  | GUDCA (stool) / GUDCA (serum) |
|  | αMUA (stool) / αMUA (serum) |
|  | TαMUA (stool) / TαMUA (serum) |
|  | GαMUA (stool) / GαMUA (serum) |
|  | βMUA (stool) / βMUA (serum) |
|  | TβMUA (stool) / TβMUA (serum) |
|  | GβMUA (stool) / GβMUA (serum) |
|  | γMUA (stool) / γMUA (serum) |
|  | ωMUA (stool) / ωMUA (serum) |
|  | TωMUA (stool) / TωMUA (serum) |
|  | HDCA (stool) / HDCA (serum) |
|  | THDCA (stool) / THDCA (serum) |
